# Mechanisms behind the Development of Chronic Low Back Pain and Its Neurodegenerative Features

**DOI:** 10.3390/life13010084

**Published:** 2022-12-28

**Authors:** Abdullah Mosabbir

**Affiliations:** 1 Neuro Spinal Innovations, Mississauga, ON L5M 1M2, Canada; amosabbir@research.baycrest.org; 2 Faculty of Music, University of Toronto, Toronto, ON M5S 2C5, Canada; 3 Baycrest Health Sciences, Rotman Research Institute, Toronto, ON M6A 2E1, Canada

**Keywords:** chronic back pain, neurodegeneration, spine, chronic pain, Alzheimer’s, aging, atrophy

## Abstract

Chronic back pain is complex and there is no guarantee that treating its potential causes will cause the pain to go away. Therefore, rather than attempting to “cure” chronic pain, many clinicians, caregivers and researchers aim to help educate patients about their pain and try to help them live a better quality of life despite their condition. A systematic review has demonstrated that patient education has a large effect on pain and pain related disability when done in conjunction with treatments. Therefore, understanding and updating our current state of knowledge of the pathophysiology of back pain is important in educating patients as well as guiding the development of novel therapeutics. Growing evidence suggests that back pain causes morphological changes in the central nervous system and that these changes have significant overlap with those seen in common neurodegenerative disorders. These similarities in mechanisms may explain the associations between chronic low back pain and cognitive decline and brain fog. The neurodegenerative underpinnings of chronic low back pain demonstrate a new layer of understanding for this condition, which may help inspire new strategies in pain education and management, as well as potentially improve current treatment.

## 1. Introduction

Low back pain is the leading cause of disability worldwide [1], with up to 80% of the population experiencing this at some point in their life [2]. The cost of back pain in the USA alone is estimated to exceed $50 billion per year [3]. Although there are many conditions that can be classified as “back pain”, this paper will not refer to back pain due to known infections, tumors, systemic diseases, fractures or dislocations. Instead, we will consider back pain with a mechanical origin that includes primary non-radicular cLBP and idiopathic cLBP.

Chronic back pain is complex and there is currently no cure for it, nor does alleviating potential causes guarantee that pain will go away. Therefore, rather than attempting to “cure” chronic pain, many clinicians, caregivers and researchers aim to help educate patients about their pain and try to help them live a better quality of life despite their condition. This may include using strategies such as pain neuroscience education (PNE) [4] and cognitive behavior therapy [5]. PNE, for example, is considered an intervention aimed at reconceptualizing an individual’s understanding of their pain as less threatening. A systematic review and meta-analysis demonstrated that PNE can have a significant effect in reducing pain catastrophizing as well as kinesiophobia [6]. This is highly beneficial in pain management, as reduced catastrophic thinking can help orient a person away from their pain and towards living their life, and reduced fear helps patients to be more open to active interventions like physical therapy and exercise. Pain education is often paired with physical therapy [7], or used pre-emptively for things like post-operative pain [8]. Educational interventions for back pain that are as short as 5 min can have a lasting impact up to 12 months [9,10,11]. These impacts can include benefits in reducing dependency of drugs, enhance self-care, facilitate behavioral change and overall improve quality of life. Therefore, understanding and updating our current state of knowledge of the pathophysiology of cLBP is important in educating patients as well as guiding the development of novel therapeutics.

Growing evidence suggests that cLBP causes morphological changes in the central nervous system and that these changes have significant overlap with those seen in common neurodegenerative disorders such as Alzheimer’s disease (AD), Parkinson’s disease (PD) and amyotropic lateral sclerosis (ALS) [12]. These similarities in mechanisms may explain the associations between cLBP and cognitive decline and brain fog [12,13]. However, the extent to which these similarities can warrant cLBP being considered a neurodegenerative disorder is not yet clear. Even if a condition is not classified as a neurodegenerative disease, identifying these features can still be beneficial by inspiring new strategies in pain education and management, as well as exploring new avenues for treatment. This review will outline a current understanding of the pathophysiology of cLBP as well as introduce evidence for a shared mechanism between cLBP and neurodegenerative disease.

## 2. The Pathophysiology of Back Pain

### 2.1. Summary of Pathophysiology

**Figure 1:**
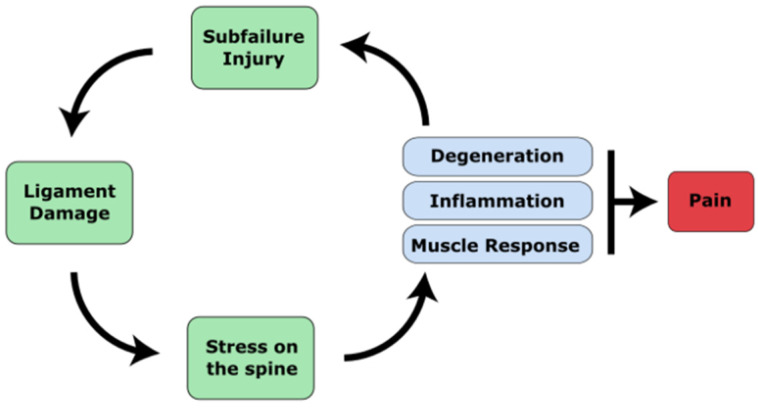
A diagram of the pathophysiology of cLBP. Major trauma or cumulative micro-trauma occurs that damages ligamentous soft tissue. Ligament damage alters the stability of the spine, which increases the risk of poor posture or spinal misalignments that introduce abnormal forces and stresses on the spine. Stress on the spine begins degeneration of the discs and joints, followed by inflammation of the discs as well as compensatory muscular responses. Pain can be caused by disc herniation or bulging that irritates nerves, nerves that grow into the disc, or degenerated facet joints that stimulates nerves. These changes, along with compensatory postures, can increase stress on the spine and further cause micro-trauma, causing pain to persist.

Chronic low back pain (cLBP) is complex and affects both the mind and the body and thus needs a multi-disciplinary approach grounded in the bio-psycho-social model [14]. Despite the importance of psycho-social factors, however, prospective studies have shown that these factors predict only 1–3% of future first-time cLBP occurrence [15]. There is no comparable evidence that psycho-social factors initiate cLBP, but instead they act to augment the experience of pain [16]. Evidence for biomechanical and physiological factors as a causative agent of cLBP, however, remain well established in the literature [16,17,18]. Therefore, to begin exploring what generates the pain in cLBP, these factors must first be outlined. In short, low cLBP begins due to spinal injury or micro-trauma that help further the degeneration of the spine, joints and associated structures. These injuries can either produce serious spinal injury, or much more commonly a type of sub-failure injury, which is defined as trauma done to the spine that is just below the threshold to produce major injury. Specifically, these can originate from three sources: (1) Muscle or ligament strain, (2) Intervertebral disc degeneration, or (3) degenerative joints. These structural changes can cause the following types of pain: myofascial pain, facet joint pain, sacroiliac joint pain, discogenic pain and spinal stenosis [19]. A summary of the pathophysiology of cLBP is illustrated in Figure 1.

### 2.2. The Ligamentous Concept

**Figure 2:**
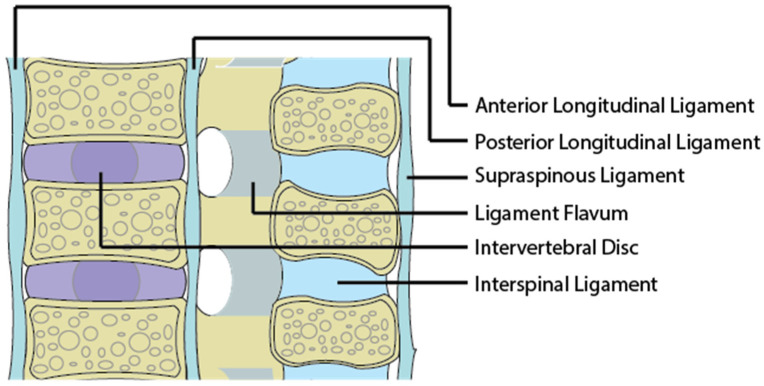
An image of a sagittal section of the spine and its associated ligaments.

The initiating source of pain from the back is due to abnormal forces acting on the body as a whole and more specifically the intervertebral discs, ligaments and facet joints. The major structural component that passively stabilizes the spine and maintain alignment are the soft tissue ligamentous structures. These include ligaments, joint capsules and intervertebral discs. Damage to these structures predisposes one to cLBP [17]. The major ligaments providing spinal stability are presented in Figure 2.

Damage to the ligaments from trauma or cumulative microtrauma can weaken the spine’s structural capabilities, leading to spinal misalignment. Trauma can occur from events such as a car accident or from repetitive movements in activities such as golfing, gymnastics and cricket [20]. Micro-trauma can also occur from poor postural habits while sitting at an office desk [17]. Abnormal mechanical stresses acting on normal ligaments can itself induce pain if it pinches a nerve. The ligament itself is innervated by neurons that can initiate a painful sensation if disturbed by mechanical or chemical irritation [21]. A misaligned spine can impose high and continuous axial load on the vertebrae, disc and facet joints. Prolonged abnormal stress on ligaments, due to misalignment, initiates degenerative and inflammatory responses that lead to pain.

### 2.3. Myofascia and Spinal Muscles

Myo-facial pain begins from trauma or repetitive motion injury [22]. This type of pain is characterized by the existence of specific points along the fascia, tendons, or muscles which, if triggered, produces pain [23]. The thoracolumbar fascia (TLF) is one structure that plays an important role in stabilizing the spine. Anatomical studies have shown that the TLF functions to transmit external loads efficiently from the spine to the pelvis, legs and arms [24,25]. The TLF also contains many mechanoreceptors that signal information on spinal position [26] and thus the TLF acts similarly to an external layer of structural support to the spine above the spinal ligaments. Other structures that support the spine are the lumbar multifidus and erector spinae. Previous studies have indicated that muscular structure changes from acute to cLBP, showing atrophy, fat infiltration and connective tissue accumulation [21,27]. These structural changes are generally explained by compensatory disuse due to changes in movement patterns to safeguard the multifidus from loads [28], pain/fear avoidance [29], or deconditioning [30]. It was reported in one study that fat infiltration into the paraspinal muscle was more relevant than disc degeneration in generating cLBP in women [31]. Another study reported that fat infiltration into the multifidus and erector spinae are highly associated with degenerative discs, which is currently the most commonly reported cause of cLBP [32]. These mechanisms serve to reduce the capability of spinal muscles in providing structural support for the spine, contributing to greater stress on the spine.

### 2.4. Spinal Muscles and Mechanoreceptors

In addition to the ligaments, spinal muscles also contribute to correct postural alignment of the spine. Once ligament damage and spinal misalignment occur, numerous mechanoreceptors located in the spinal column ligaments [33,34], facet capsules [35,36,37] and disc annulus [33] carry information to the brain. These mechanoreceptors send tactile sensations and position sense to the brain and there is evidence from animal studies in which the stimulation of ligaments [38,39], facets and discs [40] results in spinal muscle activation. Specifically, ligament fatigue [41], static flexed posture [41] and cumulative microtrauma [42] have been shown to modulate this form of spinal muscle activation. Under normal circumstances, the mechanoreceptors generate a complex signal which the brain interprets and responds with a muscle response to stabilize the spine. The damaged spine, however, behaves differently. Since mechanoreceptors are imbedded within the discs, ligaments and facet joints, damage from injury as well as degeneration may also lead to damage of these mechanoreceptors [43,44]. Damaged mechanoreceptors may send spontaneous signals to the brain regarding body position, a phenomenon referred to as ectopic mechano-sensitivity [45,46,47]. Unbalanced stresses caused by spinal muscles have been shown to induce strains to ligaments, overload facet joints [17], initiate inflammation of neural tissues [48] and accelerate disc [49] and facet joint degeneration [50]. Therefore, the overall effect of abnormal spinal muscles is to contribute to greater stress on the spine.

### 2.5. Degenerative Discs and the Inflammatory Response

**Figure 3:**
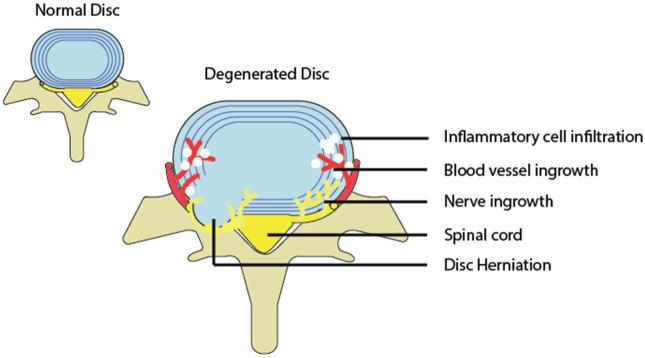
A diagram depicting a normal and degenerated disc accompanied by inflammation, as well as nerve and blood vessel growth into the disc.

Intervertebral disc degeneration is an irreversible process characterized by elevated matrix degeneration, nucleus pulposus proteoglycan loss and loss of hydration, destructuration of the disc structure and reduced disc height [51,52]. Bending loads, which can be produced by ligament damage and misalignment, can lead to disc prolapse and a cascade of cell-mediated degenerative changes. Bending mechanical stress puts extra pressure along the endplates and annulus of the disc [53], resulting in a bulge or herniation that protrudes outward. If the herniation or bulge causes mechanical compression or distension of the nerve root, dorsal root ganglion, or smaller nerves surrounding the disc, then this will lead to pain via nociceptive neural signals [54]. Further mechanical load can lead to calcification of the end plates, internal disc disruption [55,56,57] and cell-mediated loss of water content and disc height and is associated with a loss of aggrecan and collagen content within the disc [58]. Structural and material changes of the discs induce ingrowth of nerves and blood vessels within the disc [59,60], which then produces painful nerve signals (Figure 3).

### 2.6. Spinal Osteoarthritis and Degenerative Joint Disease

Osteoarthritis of the spine is a type of degenerative joint disease that is best characterized as the breakdown of cartilage in the facet joints. Ligament damage induces abnormal stress onto the facet and is a major cause of joint degeneration [61]. Degeneration of the facets can also occur as a result of degenerated discs, which shift compression loads posteriorly onto the facets. Biomechanical studies support the contribution of mechanical stresses to stimulate degeneration of the facet joints [62,63]. Furthermore, evidence from previous work has suggested that facet joint associated pain is caused by mechanical stresses induced by alignment abnormality that leads to degeneration and inflammation of the facet joints [64,65,66]. The capsule of facet joints is innervated by nociceptive neurons that can be activated by mechanical and chemical stimulation [67,68,69,70]. Direct stimulation of facet joints and ligaments can also stimulate pain [71]. Pain arising from this location is termed Facet Joint syndrome. In neurophysiologic studies using animal models, thirty mechanosensitive units were identified at the lumbar facet joint and twenty-seven were identified in the muscles and tendons near their insertion into the facet [37]. Goldthwaite et al. reported the compression of nerve roots due to a deformation of the facet joint [72]. Since then, nerve root compression by a facet joint has been considered to be one of the causes of low cLBP and sciatica [73].

## 3. The Transition from Acute to Chronic Pain

Since acute pain is tightly associated with tissue damage, it has an adaptive function as it allows one to focus on caring for the region of the body causing pain. Chronic pain can persist after the tissue damage has healed and therefore does not have an adaptive function. In some cases, chronic pain originates in the absence of any tangible injury, as defined by the international association for the study of pain (IASP) [74]. For pain to go from acute to chronic, the current literature suggests that the morphology and function of the central nervous system itself changes into a pathological state. This transition begins due to acute persistent nociceptive (i.e., pain) stimulation [75]. In peripheral nerves, persistent nociceptive stimulation produces chronic inflammation that leads to reduced pain thresholds for primary neurons, phosphorylation of protein kinases A and C, activation of TRPV1 receptors and the increased production of substance P and CGRP [76,77,78]. This situation is termed peripheral sensitization and is characterized by an increased sensitivity to afferent nerve stimuli such as heat and touch. This increased sensitivity is also called primary hyperalgesia or primary allodynia if the stimulus was not originally a painful one [79]. Although peripheral sensitization is responsible for the initial sensitization of nociceptors, this mechanism is usually short-lived, reversible and confined to a specific area of the body.

In the spinal cord, persistent nociceptive input causes changes in the dorsal root ganglion (DRG), dorsal horn neurons and glial cells. Gene and protein expression of sodium and TRPV1 channels increase in the DRG, leading to more excitable neurons [80]. NMDA receptors in the dorsal horn neurons also become more excitable, leading to the ‘wind-up’ phenomenon. Wind-up refers to an increase in pain intensity over time; when a given stimulus is delivered repeatedly and clinically, this manifests as allodynia. Prolonged nociceptive transmission to the spinal cord also activates glial cells by increasing neuronal chemokines, neurotransmitters and neuromodulators and produces endogenous danger signals [78,81]. Once activated, glial cells release substances into the central nervous system (IL-1, IL-6, TNF, chemokines, prostaglandins, excitatory amino acids, reactive oxygen species and nitric oxide), which has the cumulative effect of enhancing neuronal excitability [82,83]. This results in central sensitization, which is a state of reduced thresholds to stimulus associated with the activation of neurons in the spinal cord [76]. This type of sensitization produces secondary hyperalgesia, is long-term, and the pain can spread to other parts of the body not associated with injury.

Pain and temperature are sensed and relayed to the brain via the lateral spinothalamic tract, while crude tactile sensation travels through the anterior spinothalamic tract. Nociceptive signals travel through free nerve endings into the dorsal horn of the spinal cord and progress upward to the thalamus of the brain. The thalamus is responsible for collecting all sensory information and filtering it, only passing information forward upon summation of multiple signals over a threshold. The signal is then sent to multiple cortical areas for interpretation. Disruption in these thalamocortical projections is thought to play an important role in pain perception and also in neuropathic pain. Abnormal oscillatory activity in thalamo–cortico–thalamo loops, termed thalamocortical dysrhythmia, have become a hallmark for chronic conditions such as chronic pain [84]. The transition from acute to chronic pain involves continuous nociceptive input to the brain which alters both brain anatomy and function over time. For example, acute back pain triggers the insular cortex for pain perception, which projects strongly to the nucleus accumbans (NAcc), increasing motivational behaviour in response to pain. As acute pain transitions to chronic pain, the connection between the IS and NAcc decreases, while the connection between the NAcc and medial prefrontal cortex (mPFC) increases. The mPFC is known for processing negative emotions, response conflict and the detection of unfavourable outcomes [85]. Greater pain was also associated with reduced parietal activity corresponding to reduced attentional processing during visual tasks [86]. Other reports have also confirmed that the resting state activity of the brain is distorted in chronic pain conditions [87,88,89,90]. These disruptions of brain activity in chronic pain patients have been shown to produce metabolic changes that lead to degeneration of prefrontal and thalamic grey matter in the brain [91,92]. Further brain imaging studies for chronic pain have demonstrated that chronic pain consistently engages the mPFC as well as subcortical limbic areas, including the portions of the dorsal and ventral basal ganglia [93], amygdala [94] and hippocampus [95]. These areas of the brain are responsible for motivation, emotional processing and memory. Therefore, even though different types of chronic pain show a unique activation of brain areas, the transition from acute to chronic pain consistently demonstrates a shift away from purely sensory brain processing towards emotional, motivational and memory processing. These changes may explain observed behaviors such as pain catastrophizing and fear-avoidance.

## 4. Neurodegenerative Changes in the Brain

The National Cancer Institute defined a neurodegenerative disorder as one in which the cells of the central nervous system stop working or die. Apoptosis is a form of programmed cell death that occurs in neurodegenerative disorders such as Alzheimer’s, Parkinson’s, Huntington’s disease, Amyotrophic lateral sclerosis (ALS) and stroke. Triggers for apoptosis of neurons in these conditions include amyloid b-peptide, aggregates of Tau filaments, oxidative or metabolic stress, excess levels of neurotransmitter glutamate and death receptor activation by cytokines [96,97]. Animal models of chronic neuropathic pain have demonstrated apoptotic cell death in the dorsal horn where nociceptors terminate [98,99]. This form of degeneration is slow and long lasting. Spared nerve injury models of rats exhibiting allodynia and hyperalgesia showed the neurodegeneration of dorsal horn interneurons that modulate nociceptive sensory input [100,101,102]. In these chronic pain models, the loss of such interneurons was directly associated with a decrease of inhibitory control of nociceptive signals and an increase in pain sensitivity [103,104,105]. Apoptosis of these neurons are triggers by afferent nerve activity, as the blockage of afferent nerve signals with bupivacaine reduced the number of apoptotic profiles in the dorsal horn and this lasted as long as the nerve block persisted [102]. Therefore, the current theory is that persistent pain signals, which transition pain from acute to chronic through a process of peripheral and central sensitization, may also provide the afferent activity necessary to trigger the neurodegeneration of spinal cord neurons.

### 4.1. Brain Metabolites and Grey Matter Changes in cLBP

In the neurodegenerative brain, measuring the brain metabolite N-acetyl-aspartate (NAA) has become a well-accepted tool to measure neuronal density and therefore neurodegeneration, among disorders such as Alzheimer’s and Parkinson’s [12]. These brain metabolites are typically much lower in neurodegenerative conditions and have been shown to correlate strongly with disease progression of Alzheimer’s [106,107,108,109]. NAA has been found to be decreased in the dorsolateral prefrontal cortex (DLPFC) of those with cLBP and this correlated with the pain intensity as well as the affective dimensions of cLBP [110]. A follow up study showed that the NAA concentration in the DLPFC and orbitofrontal cortex was highly correlated to pain assessment scores from the McGill Pain Questionnaire [111]. The association of pain assessment scores and decreased levels of NAA in the thalamus has also been observed in patients with central pain after spinal cord injury and with complex regional pain syndrome or postherpetic neuralgia [112,113]. Decreased concentrations of other brain metabolites such as glucose or inositol have been found in the cerebrospinal fluid of patients with cLBP related to a herniated disc or spinal stenosis [114]. MRI images of the brain of those with cLBP supported these metabolite based findings by demonstrating a reduction in grey matter within the DLPFC and thalamus, indicating that neurodegeneration of these structures a key feature of cLBP. A recent meta-analysis comprising 10 studies of 293 patients with cLBP and 624 healthy controls concluded that regional grey matter abnormalities was found in areas from the bilateral medial prefrontal cortex extending to the anterior cingulate cortex and the right media prefrontal cortex extending to the orbitofrontal cortex [115]. The total volume of grey matter was negatively correlated with the duration of pain, such that for every year lived with cLBP, there is a decrease in cortical volume by 1.5 cm^3^ more than that associated with aging. One study looking at regional brain atrophy via MRI imaging of 1106 elderly adults found that compared with matched controls without cLBP, those with cLBP had significantly lower brain volumes in the area of ventrolateral and dorsolateral prefrontal cortex, posterior cingulate gyrus and the amygdala [116]. Neurodegeneration of specific brain regions has also been found in other chronic pain states [12,117].

### 4.2. Cognitive Impairment in cLBP

**Figure 4:**
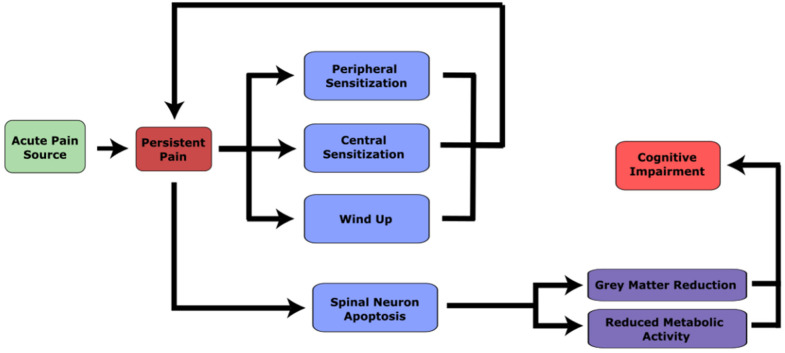
A diagram illustrating the transition from acute to chronic pain and the development of neurodegenerative features. An acute source of pain that persists triggers neuroplastic changes in the central nervous system. This produces peripheral sensitization, central sensitization and wind up symptoms. These in turn generate pain throughout the body that is independent of the source of the acute pain. Persistent pain is also associated with spinal neuron degeneration, followed by brain grey matter reduction and reduced metabolic activity leading to cognitive impairment.

One of the clinical consequences of brain abnormalities among cLBP patients is cognitive impairment (Figure 4). Neurodegenerative disorders often get worse over time and are therefore highly associated with the aging process. Based on brain grey matter density, one study estimated brain age using a machine learning technique and found that those with non-depressed cLBP demonstrated accelerated brain aging compared to healthy controls [13,118]. People with chronic pain typically perform worse on measures of global cognition than healthy controls [119] and the prevalence of cognitive impairment among cLBP patients is also greater [120,121]. Impaired cognitive functions have been demonstrated across different domains such as memory and executive function, verbal working memory, non-verbal working memory and attention [122]. Compared with normative data, those with cLBP have been shown to have deficits in verbal, visual and spatial memory [123]. There are several theories about why cognition suffers as cLBP develops, most of which involve the DLPFC, an area important for cognitive functions such as executive function, planning skills and memory [122,124]. One theory suggests that the processing of nociceptors engages a significant amount of resources of the DLPFC, making it less effectively used for cognitive functions [124]. Another theory is that decreased cortical inhibition extends the activity of DLPFC, which again uses resources otherwise allocated for cognitive functions [122]. Changes in the attention and default mode network also require cognitive resources that can influence cognitive function [125,126]. Based on the neurodegenerative mechanisms summarized in this paper, we can add a third theory: the brain related changes in grey matter and metabolism characteristic of ‘neurodegeneration’ among cLBP patients may contribute to cognitive decline among those with chronic pain. It is not clear if the degree to which the relationship between cLBP and cognitive impairment is associative, or whether one strictly causes another, but this paper highlights one pathway for cognitive impairment to develop after cLBP.

### 4.3. Is cLBP a Neurodegenerative Disorder?

Although grey matter reduction is highly indicative of a neurodegenerative mechanism, it is still unclear if cLBP should be considered as a neurodegenerative disease. Grey matter reduction in cLBP is potentially reversible with successful treatment. A research group in McGill found that, after 6 months of treatment of facet joint injections, cLBP patients that improved in their pain symptoms showed greater cortical thickness in the DLPFC as well as their motor cortex [127]. Other studies showed that successful pain relief after surgery for chronic pain due to unilateral coxarthrosis yielded a subsequent grey matter increase in the anterior cingulate cortex, the DLPFC and orbitofrontal cortex [126,128]. Although this may lead some to suggest that brain related changes due to chronic pain may not be neurodegenerative, this assertion has limitations. First, this makes the assumption that neurodegeneration is definitively irreversible. Animal models for Alzheimer’s, Parkinson’s and Huntington’s disease have shown promise in reversing neurodegenerative effects when pathological cellular pathways are inhibited [129,130,131]. Secondly, reversals in grey matter seen in chronic pain cases are highly dependent on successful treatment of chronic pain, which in practice is not always easy to achieve. Pain that transitions to a chronic state cannot be cured simply by treating the cause and thus may persist indefinitely. By the time a clinician sees a patient complaining about chronic pain, the “cause” of that pain may have long healed, making the diagnosis and treatment of such a condition very difficult. In such a situation these mechanisms may otherwise be considered irreversible, which is not too different from our current predicament in treating neurodegenerative disorders. Thirdly, it is also not shown whether neurodegeneration of the spinal cord, apoptosis of neurons, or reduced brain metabolites as seen in cLBP can be reversed with successful treatment. Therefore, the current evidence suggests that the mechanism of cLBP can be relatable to that of neurodegenerative conditions.

The value in understanding cLBP as having neurodegenerative features is in developing better patient education strategies and exploring novel options for treating cLBP. Baliki and Apkarian suggested that cLBP should be considered as a neurological or neurodegenerative condition and that targeting these neurological complications can help treat pain [12,132]. One suggestion is the use of neurological drugs targeting the excitotoxic death of dorsal root neurons, reduced inhibition, or microglia activation. These would include a range of drugs such as N-methyl-D-aspartate (NDMA) antagonists, gamma-aminobutyric acid (GABA) agonists and Caspase inhibitors [12]. Another strategy may be cognitive behaviour therapy (CBT), which has shown promise for cLBP. CBT can influence functional and metabolic activity of brain regions in a chronic pain state [133]. One study aimed to measure brain network activity after repeated pain stimulus with and without CBT. They found that those with CBT did not demonstrate the neurotypical decrease in default mode network activity seen with persistent pain and this was linked to reduced pain intensity and unpleasantness over time [134]. A third avenue may be neuromodulation, which is a technique used for Alzheimer’s and Parkinson’s that may also work for chronic pain. Neuromodulation aims to recover lost brain function by stimulating the oscillatory activity of specific brain regions [135]. One example of an emerging treatment modality is the use of vibratory stimuli as a form of neuromodulation, which has shown promise in treating symptoms of Alzheimer’s [136,137], Parkinson’s [138,139] and depressive disorders [140,141]. Vibratory stimuli, especially those focused on stimulating the spine, have also emerged as a promising new treatment modality when applied to treat cLBP [142,143,144,145]. These waveforms have also demonstrated changes in proteoglycan expression within the intervertebral discs, showing a possible mechanistic influence on spinal structures [146,147]. Although it is still not clear if cLBP can be considered a neurodegenerative condition, understanding its neurodegenerative features can open new doors for the management and treatment of cLBP.

## 5. Conclusions

cLBP is complex and there is no guarantee that treating its potential causes will cause the pain to go away. Therefore, rather than attempting to “cure” chronic pain, many clinicians, caretakers and researchers aim to help educate patients about their pain and try to help patients live a better quality of life, despite their condition. Understanding and updating our current state of knowledge of the pathophysiology of cLBP is important in educating patients as well as guiding the development of novel therapeutics.

The current understanding of the source of cLBP stems from damage to the ligamentous structures of the body due to prolonged poor posture, sub-failure injury, or major trauma. These contribute to abnormal forces acting on the spine, which leads to the degeneration of intervertebral discs and joints that directly or indirectly stimulate a painful sensation. If these sensations persist, acute pain transitions to a chronic state that sustains the pain independent of the source of the initial damage. Once this occurs, further degeneration occurs in the spinal cord and brain, leading to peripheral sensitization, central sensitization, apoptosis of neurons and the reduction of grey matter in the brain. This ultimately can lead to cognitive impairment among cLBP patients. Growing evidence has indicated that these mechanisms have a shared biology with neurodegenerative conditions; however, further evidence is needed to know whether these mechanisms can warrant cLBP being considered a neurodegenerative condition. Despite this, however, our understanding of the neurodegenerative features of cLBP can improve our efforts to understand and treat cLBP. Cognitive difficulties among cLBP patients should be identified and addressed more seriously in clinical settings. In addition, medications targeting cognitive impairment may provide benefit to patients with pain, as was proposed in a previous review [12]. Therefore, the neurodegenerative underpinnings of cLBP uncover a new layer of understanding for this condition, which may help inspire new strategies in pain management or may improve current treatment strategies.

## Data Availability

Not applicable.
